# Supplementary material for: Cryo-EM structure of the extracellular domain of murine Thrombopoietin Receptor in complex with Thrombopoietin
Source: Nat Commun. 2024 Feb 7;15:1135. doi: 10.1038/s41467-024-45356-2 (PMC10850085; doi:10.1038/s41467-024-45356-2)
Supplement: Supplementary file 1 — Supplementary Information [file 41467_2024_45356_MOESM1_ESM.pdf]

Supplementary Information

Cryo-EM structure of the extracellular domain of murine Thrombopoietin Receptor in complex with Thrombopoietin.

Kaiseal T. G. Sarson-Lawrence[1,2,5], Joshua M. Hardy[1,2,3,5], Josephine Iaria[1], Dina Stockwell[1,2], Kira Behrens[1,2], Tamanna Saiyed[1,2], Cyrus Tan[1,2], Leila Jebeli[4], Nichollas E. Scott[4], Toby A. Dite[1,2], Nicos A. Nicola[1,3], Andrew P. Leis[1,2,3], Jeffrey J. Babon[1,2†] and Nadia J. Kershaw[1,2†]

1. Walter and Eliza Hall Institute of Medical Research, 1G Royal Parade, Parkville, 3052, Victoria, Australia

2. Department of Medical Biology, The University of Melbourne, Royal Parade, Parkville, 3052, Victoria, Australia

3. ARC Centre for Cryo-electron Microscopy of Membrane Proteins, Walter and Eliza Hall Institute of Medical Research, 1G Royal Parade, Parkville, 3052, Victoria, Australia

4. Department of Microbiology and Immunology, University of Melbourne at the Peter Doherty Institute for Infection and Immunity, Melbourne 3000, Australia

[†]Authors for correspondence: Babon@wehi.edu.au, Kershaw@wehi.edu.au.
[5]These authors contributed equally: Kaiseal T.G. Sarson-Lawrence, Joshua M. Hardy

Supplementary Table 1 - Sequences of proteins used in this study.

**Protein**	**Sequence**
TpoR$_{1-482}$-LeuZ	*TpoRsignalpeptide*-TpoR$_{26-482}$-LeuZ-linker-*TEV*-linker-Fc-FLAG<br>(blue=sample for cryo-EM)<br>*MPSWALFMVTSCLLLALPNQAQVT*SQDVFLLALGTEPLNCFSQTFEDLTCFWDEEEAAPSGTYQLL<br>YAYRGEKPRACPLYSQSVPTFGTRYVCQFPAQDEVRLFFPLHLWVKNVSLNQTLIQRVLFVDSVGLP<br>APPRVIKARGGSQPGELQIHWEAPAPEISDFLRHELRYGPTDSSNATAPSVIQLLSTETCCPTLWMP<br>NPVPVLDQPPCVHPTASQPHGPAPFLTVKGGSCLVSGLQAGKSYWLQLRSQPDGVSLRGSWGP<br>WSFPVTVDLPGDAVTIGLQCFTLDLKMVTCQWQQQDRTSSQGFFRHSRTRCCPTDRDPTWEKCE<br>EEEPRPGSQPALVSRCHFKSRNDSVIHILVEVTTAQGAVHSYLGSPFWIHQAVLLPTPSLHWREVSS<br>GRLELEWQHQSSWAAQETCYQLRYTGEGREDWKVLEPSLGARGGTLELRPRARYSLQLRARLNGP<br>TYQGPWSAWSPPARVSTGSETAWRMKQLEDKVEELLSKNYHLENEVARLKKLVGERTGGAP*ENL*<br>*YFQ*GSSGGPSVFLFPPKPKDTLMISRTPEVTCVVVDVSHEDPEVKFNWYVDGVEVHNAKTKPREE<br>QYNSTYRVVSVLTVLHQDWLNGKEYKCKVSNKALPAPIEKTISKAKGQPREPQVYTLPPSRDELTKN<br>QVNLTCLVKGFYPSDIAVEWESNGQPENNYKTTPPVLDSDGSFFLNSTLTVDKSRWQQGNVFSCS<br>VMHEALHNHYTQKSLSLSPGKDYKDDDDK
TpoN$_{22-184}$	*mIL3SignalPeptide*-linker-FLAG-*linker*-Tpo$_{22-184}$<br>(blue = sample for cryo-EM)<br>*MVLASSTTSIHTMLLLLLMLFHLGLQASIS*ARQDYKDDDDK*TRQ*SPVAPACDPRLLNKLLRDSHLLH<br>SRLSQCPDVDPLSIPVLLPAVDFSLGEWKTQTEQSKAQDILGAVSLLLEGVMAARGQLEPSCLSSLL<br>GQLSGQVRLLLGALQGLLGTQLPLQGRTTAHKDPNALFLSLQQLLRGKVRFLLLVEGPTLCVRRTLP<br>TTAVPS
TpoR$_{1-479}$	*TpoRSignalPeptide*-TpoR$_{1-479}$-*TEV*-Fc-FLAG<br>*MPSWALFMVTSCLLLALPNQAQVT*SQDVFLLALGTEPLNCFSQTFEDLTCFWDEEEAAPSGTYQLL<br>YAYRGEKPRACPLYSQSVPTFGTRYVCQFPAQDEVRLFFPLHLWVKNVSLNQTLIQRVLFVDSVGLP<br>APPRVIKARGGSQPGELQIHWEAPAPEISDFLRHELRYGPTDSSNATAPSVIQLLSTETCCPTLWMP<br>NPVPVLDQPPCVHPTASQPHGPAPFLTVKGGSCLVSGLQAGKSYWLQLRSQPDGVSLRGSWGP<br>WSFPVTVDLPGDAVTIGLQCFTLDLKMVTCQWQQQDRTSSQGFFRHSRTRCCPTDRDPTWEKCE<br>EEEPRPGSQPALVSRCHFKSRNDSVIHILVEVTTAQGAVHSYLGSPFWIHQAVLLPTPSLHWREVSS<br>GRLELEWQHQSSWAAQETCYQLRYTGEGREDWKVLEPSLGARGGTLELRPRARYSLQLRARLNGP<br>TYQGPWSAWSPPARVSTGSETAWGGAP*ENLYFQG*SSGGPSVFLFPPKPKDTLMISRTPEVTCVVV<br>DVSHEDPEVKFNWYVDGVEVHNAKTKPREEQYNSTYRVVSVLTVLHQDWLNGKEYKCKVSNKAL<br>PAPIEKTISKAKGQPREPQVYTLPPSRDELTKNQVNLTCLVKGFYPSDIAVEWESNGQPENNYKTTP<br>PVLDSDGSFFLNSTLTVDKSRWQQGNVFSCSVMHEALHNHYTQKSLSLSPGKDYKDDDDK
Tpo	*mIL3SignalPeptide*-linker-FLAG-*linker*-Tpo$_{22-356}$<br>*MVLASSTTSIHTMLLLLLMLFHLGLQASIS*ARQDYKDDDDK*TRQ*SSPVAPACDPRLLNKLLRDSHLL<br>HSRLSQCPDVDPLSIPVLLPAVDFSLGEWKTQTEQSKAQDILGAVSLLLEGVMAARGQLEPSCLSSL<br>LGQLSGQVRLLLGALQGLLGTQLPLQGRTTAHKDPNALFLSLQQLLRGKVRFLLLVEGPTLCVRRTL<br>PTTAVPSSTSQLLTLNKFPNRTSGLLETNFSVTARTAGPGLLSRLQGFRVKITPGQLNQTSRSPVQIS<br>GYLNRTHGPVNGTHGLFAGTSLQTLEASDISPGAFNKGSLAFNLQGGLPPSPSLAPDGHTPFPPSP<br>ALPTTHGSPPQLHPLFPDPSTTMPNSTAPHPVTMYPHPRNLSQET
TpoR$_{1-482}$-LeuZ<br>Mutant 1	*TpoRsignalpeptide*-TpoR$_{26-482}$-LeuZ-linker-*TEV*-Fc-FLAG<br>*MPSWALFMVTSCLLLALPNQAQVT*SQDVFLLALGTEPLNCFSQTFEDLTCFWDEEEAAPSGTYQLL<br>YAYRGEKPRACPLYSQSVPTFGTRYVCQFPAQDEVRLFFPLHLWVKNVSLNQTLIQRVLFVDSVGLP<br>APPRVIKARGGSQPGELQIHWEAPAPEISDFLRHELRYGPTDSSNATAPSVIQLLSTETCCPTLWMP<br>NPVPVLDQPPCVHPTASQPHGPAPFLTVKGGSCLVSGLQAGKSYWLQLRSQPDGVSLRGSWGP<br>WSFPVTVDLPGDAVTIGLQCFTLDLKMVTCQWQQQDRTSSQGFFRHSRTRCCPTDRDPTWEKCE<br>EEEPRPGSQPALVSRCHFKSRNDSVIHILVEVTTAQGAVHSYLGSPFWIHQAVLLPTPSLHWREVSS<br>GRLELEWQHQSSWAAQETCYQLRYTGEGREDWKVLEPSLGARGGTLELRPRARYSLQLRARLNGP<br>TYQGPWSAWSPPARVSTGSERMKQLEDKVEELLSKNYHLENEVARLKKLVGERTGGLNDIFEAQKI

	EWHETGGAP*ENLYFQG*SSGGPSVFLFPPKPKDTLMISRTPEVTCVVVDVSHEDPEVKFNWYVDGV<br>EVHNAKTKPREEQYNSTYRVVSVLTVLHQDWLNGKEYKCKVSNKALPAPIEKTISKAKGQPREPQV<br>YTLPPSRDELTKNQVNLTCLVKGFYPSDIAVEWESNGQPENNYKTTPPVLDSDGSFFLNSTLTVDKS<br>RWQQGNVFSCSVMHEALHNHYTQKSLSLSPGKDYKDDDDK
TpoR$_{1-482}$-LeuZ<br>Mutant 2	*TpoRsignalpeptide*-TpoR$_{26-482}$-LeuZ-linker-*TEV*-Fc-FLAG<br>*MPSWALFMVTSCLLLALPNQAQVT*SQDVFLLALGTEPLNCFSQTFEDLTCFWDEEEAAPSGTYQLL<br>YAYRGEKPRACPLYSQSVPTFGTRYVCQFPAQDEVRLFFPLHLWVKNVSLNQTLIQRVLFVDSVGLP<br>APPRVIKARGGSQPGELQIHWEAPAPEISDFLRHELRYGPTDSSNATAPSVIQLLSTETCCPTLWMP<br>NPVPVLDQPPCVHPTASQPHGPAPFLTVKGGSCLVSGLQAGKSYWLQLRSQPDGVSLRGSWGP<br>WSFPVTVDLPGDAVTIGLQCFTLDLKMVTCQWQQQDRTSSQGFFRHSRTRCCPTDRDPTWEKCE<br>EEEPRPGSQPALVSRCHFKSRNDSVIHILVEVTTAQGAVHSYLGSPFWIHQAVLLPTPSLHWREVSS<br>GRLELEWQHQSSWAAQETCYQLRYTGEGREDWKVLEPSLGARGGTLELRPRARYSLQLRARLNGP<br>TYQGPWSAWSPPARVSTGSEQRMKQLEDKVEELLSKNYHLENEVARLKKLVGERTGGLNDIFEAQ<br>KIEWHETGGAP*ENLYFQG*SSGGPSVFLFPPKPKDTLMISRTPEVTCVVVDVSHEDPEVKFNWYVD<br>GVEVHNAKTKPREEQYNSTYRVVSVLTVLHQDWLNGKEYKCKVSNKALPAPIEKTISKAKGQPREP<br>QVYTLPPSRDELTKNQVNLTCLVKGFYPSDIAVEWESNGQPENNYKTTPPVLDSDGSFFLNSTLTVD<br>KSRWQQGNVFSCSVMHEALHNHYTQKSLSLSPGKDYKDDDDK
TpoR$_{1-482}$-LeuZ<br>Mutant 3	*TpoRsignalpeptide*-TpoR$_{26-482}$-LeuZ-linker-*TEV*-Fc-FLAG<br>*MPSWALFMVTSCLLLALPNQAQVT*SQDVFLLALGTEPLNCFSQTFEDLTCFWDEEEAAPSGTYQLL<br>YAYRGEKPRACPLYSQSVPTFGTRYVCQFPAQDEVRLFFPLHLWVKNVSLNQTLIQRVLFVDSVGLP<br>APPRVIKARGGSQPGELQIHWEAPAPEISDFLRHELRYGPTDSSNATAPSVIQLLSTETCCPTLWMP<br>NPVPVLDQPPCVHPTASQPHGPAPFLTVKGGSCLVSGLQAGKSYWLQLRSQPDGVSLRGSWGP<br>WSFPVTVDLPGDAVTIGLQCFTLDLKMVTCQWQQQDRTSSQGFFRHSRTRCCPTDRDPTWEKCE<br>EEEPRPGSQPALVSRCHFKSRNDSVIHILVEVTTAQGAVHSYLGSPFWIHQAVLLPTPSLHWREVSS<br>GRLELEWQHQSSWAAQETCYQLRYTGEGREDWKVLEPSLGARGGTLELRPRARYSLQLRARLNGP<br>TYQGPWSAWSPPARVSTGSELQRMKQLEDKVEELLSKNYHLENEVARLKKLVGERTGGLNDIFEA<br>QKIEWHETGGAP*ENLYFQG*SSGGPSVFLFPPKPKDTLMISRTPEVTCVVVDVSHEDPEVKFNWYV<br>DGVEVHNAKTKPREEQYNSTYRVVSVLTVLHQDWLNGKEYKCKVSNKALPAPIEKTISKAKGQPRE<br>PQVYTLPPSRDELTKNQVNLTCLVKGFYPSDIAVEWESNGQPENNYKTTPPVLDSDGSFFLNSTLTV<br>DKSRWQQGNVFSCSVMHEALHNHYTQKSLSLSPGKDYKDDDDK
TpoR$_{1-482}$-LeuZ<br>Mutant 4	*TpoRsignalpeptide*-TpoR$_{26-482}$-LeuZ-linker-*TEV*-Fc-FLAG<br>*MPSWALFMVTSCLLLALPNQAQVT*SQDVFLLALGTEPLNCFSQTFEDLTCFWDEEEAAPSGTYQLL<br>YAYRGEKPRACPLYSQSVPTFGTRYVCQFPAQDEVRLFFPLHLWVKNVSLNQTLIQRVLFVDSVGLP<br>APPRVIKARGGSQPGELQIHWEAPAPEISDFLRHELRYGPTDSSNATAPSVIQLLSTETCCPTLWMP<br>NPVPVLDQPPCVHPTASQPHGPAPFLTVKGGSCLVSGLQAGKSYWLQLRSQPDGVSLRGSWGP<br>WSFPVTVDLPGDAVTIGLQCFTLDLKMVTCQWQQQDRTSSQGFFRHSRTRCCPTDRDPTWEKCE<br>EEEPRPGSQPALVSRCHFKSRNDSVIHILVEVTTAQGAVHSYLGSPFWIHQAVLLPTPSLHWREVSS<br>GRLELEWQHQSSWAAQETCYQLRYTGEGREDWKVLEPSLGARGGTLELRPRARYSLQLRARLNGP<br>TYQGPWSAWSPPARVSTGSEKLQRMKQLEDKVEELLSKNYHLENEVARLKKLVGERTGGLNDIFE<br>AQKIEWHETGGAP*ENLYFQG*SSGGPSVFLFPPKPKDTLMISRTPEVTCVVVDVSHEDPEVKFNWY<br>VDGVEVHNAKTKPREEQYNSTYRVVSVLTVLHQDWLNGKEYKCKVSNKALPAPIEKTISKAKGQPR<br>EPQVYTLPPSRDELTKNQVNLTCLVKGFYPSDIAVEWESNGQPENNYKTTPPVLDSDGSFFLNSTLT<br>VDKSRWQQGNVFSCSVMHEALHNHYTQKSLSLSPGKDYKDDDDK

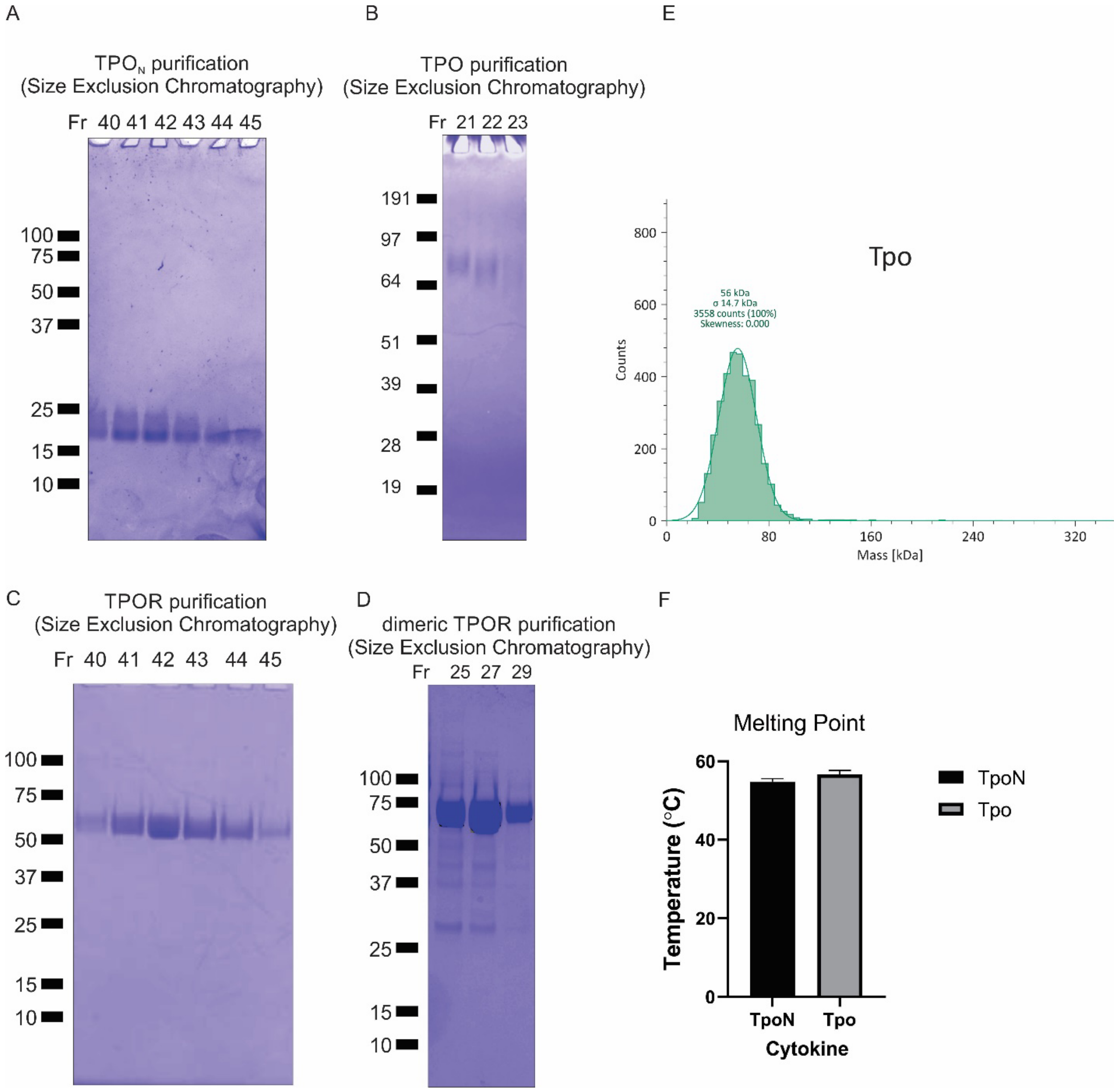

Supplementary Figure 1. Protein Purification and characterisation.

(A) Coomassie stained gel of a TpoN purification. Individual lanes are fractions from a Gel Filtration purification. (B) Coomassie stained gel of a Tpo purification. Individual lanes are fractions from a Gel Filtration purification. (C) Coomassie stained gel of a TpoR purification. Individual lanes are fractions from a Gel Filtration purification. (D) Coomassie stained gel of a dimeric TpoR purification. Individual lanes are fractions from a Gel Filtration purification. (E) Mass-photometric analysis of Tpo indicates a molecular weight of 56 kDa (Theoretical MW without glycosylation = 39 kDa). (F) Nano-DSF analysis of the melting point of TpoN and Tpo.

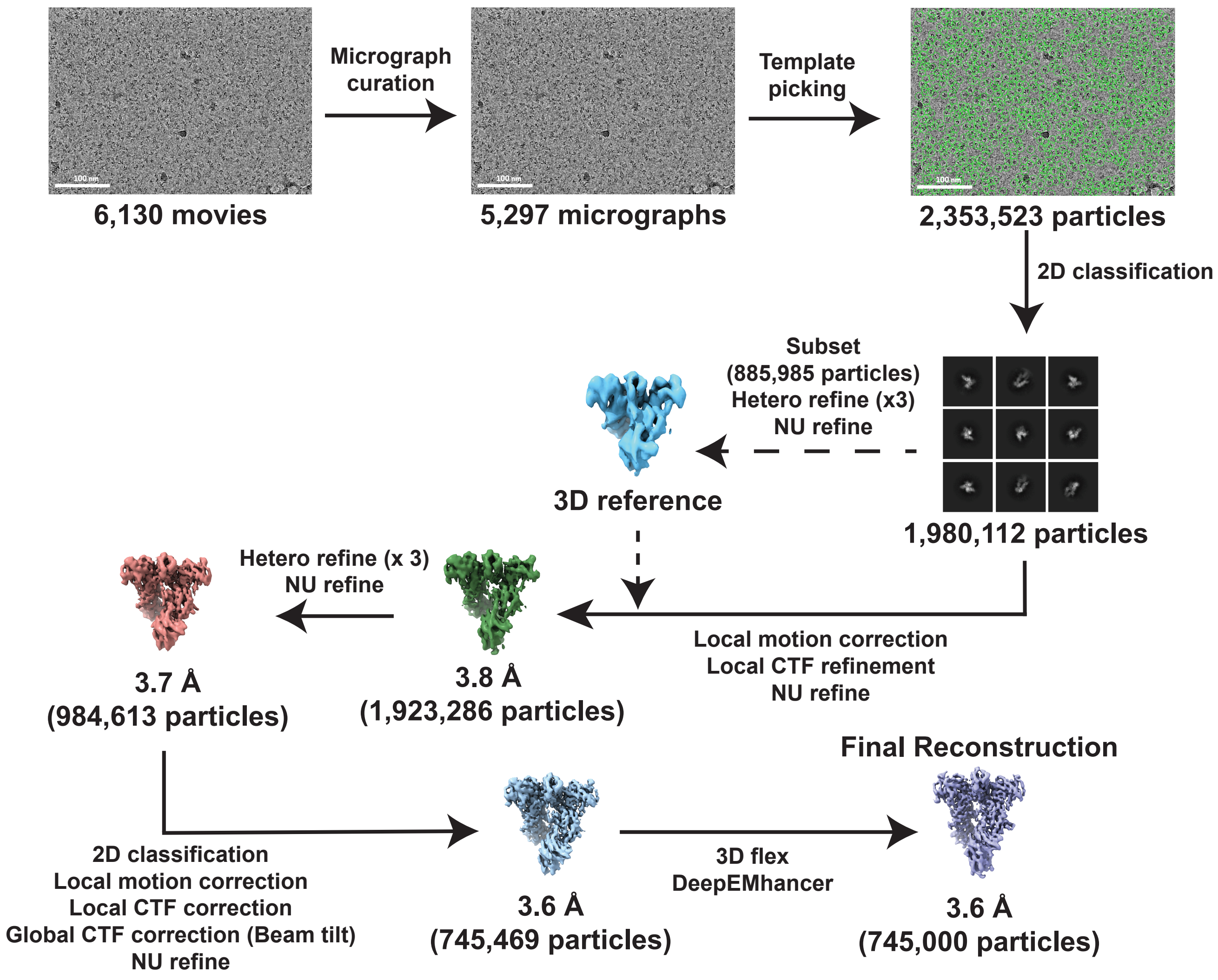

Supplementary Figure 2. Overview of cryo-EM data processing workflow in cryoSPARC. After the first 2D classification, a subset of the data was used to generate a 3D reference which was used to perform local motion correction and CTF correction of the entire dataset.

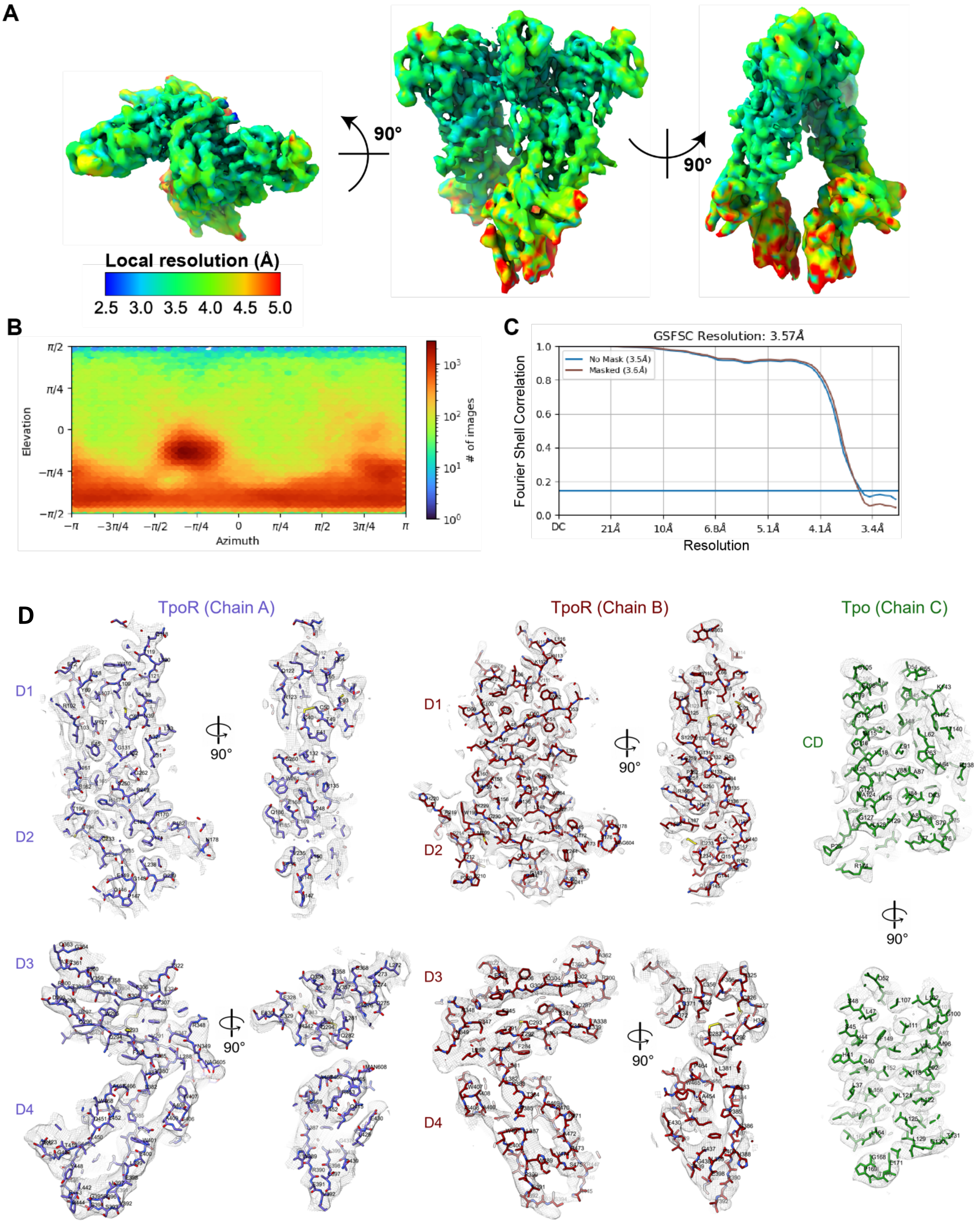
A
90°
90°
Local resolution (Å)
2.5 3.0 3.5 4.0 4.5 5.0
B
Elevation
Azimuth
# of images
C
GSFSC Resolution: 3.57Å
No Mask (3.5Å)
Masked (3.6Å)
Fourier Shell Correlation
Resolution
D
TpoR (Chain A)
TpoR (Chain B)
Tpo (Chain C)
D1
D2
D3
D4
CD
90°

Supplementary Figure 3. Qualitative assessment of cryo-EM reconstruction.

(A) Reconstruction coloured according to the local resolution. (B) Orientation distribution plot of particles used in refinement. (C) Gold-standard Fourier shell correlation (GSFSC) plots of the final 3DFlex refinement. (D) Cut-away views of the cryo-EM map (grey mesh) depicting the fit of the TpoR:Tpo model (stick representation). The cryo-EM map is contoured at 4σ and zoned within 3.5 Å of modelled atoms.

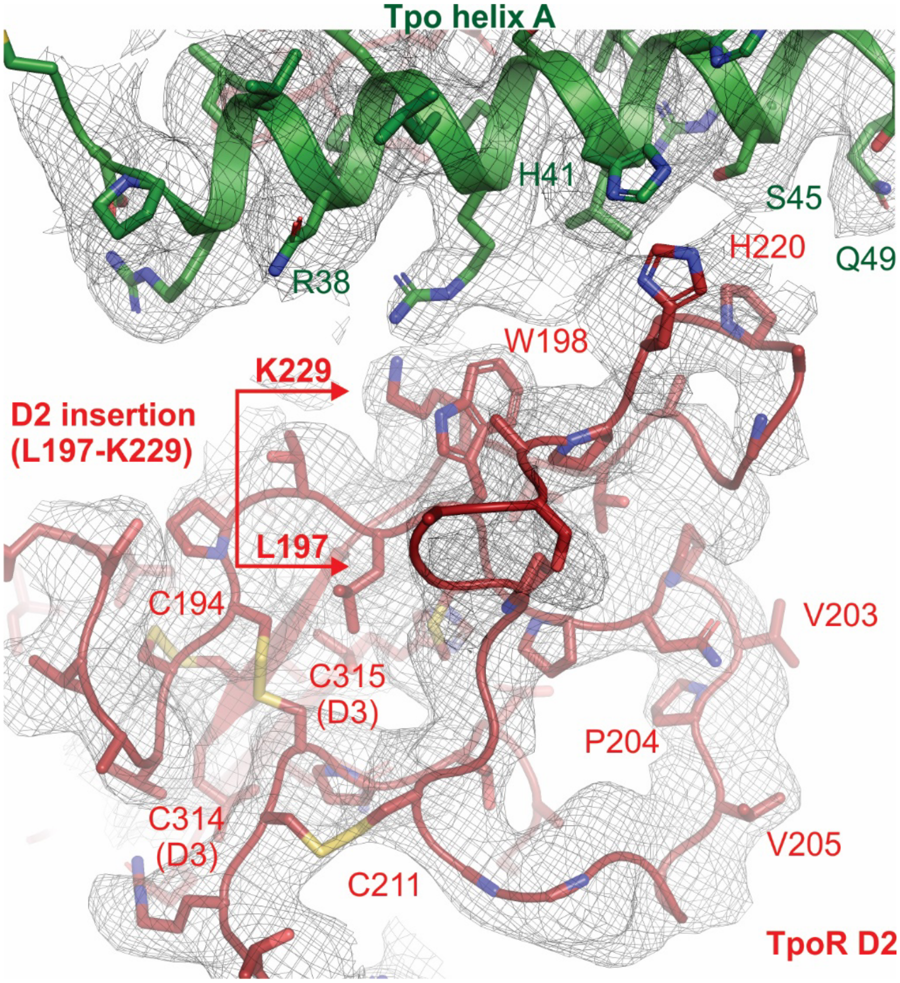

Supplementary Figure 4. Site IIb. The interaction between the D2 insertion and Tpo is shown. The D2 insertion runs from N196-K229 and is only visible in chain B. This region is shown in cartoon and stick representation [Tpo (green), TpoR(red)] with the EM map contoured at 3σ. Although there is near-continuous density for the entire insertion it is poorly resolved and many of the sidechain orientations are ambiguous. The loop interacts with Tpo helix A (top of figure) and TpoR domain 3 (D3) to which it is disulphide bonded (bottom left of figure).

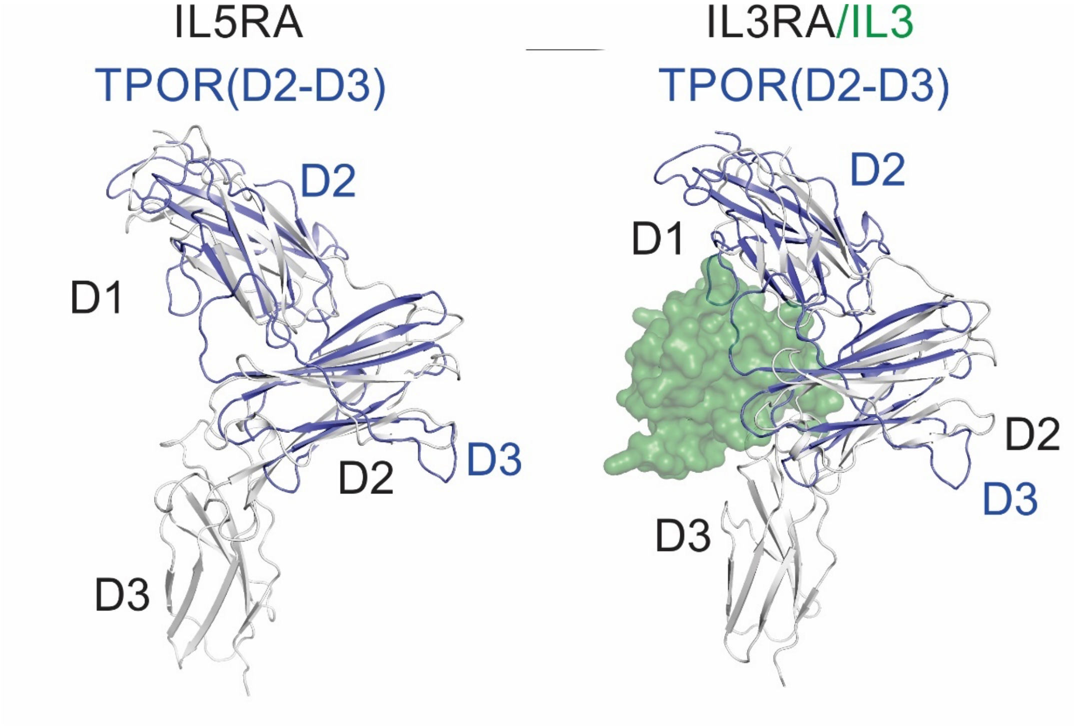

Supplementary Figure 5. Overlay of the TpoR D2-D3 interface (blue) with the IL-3 and IL-5 receptors (white).

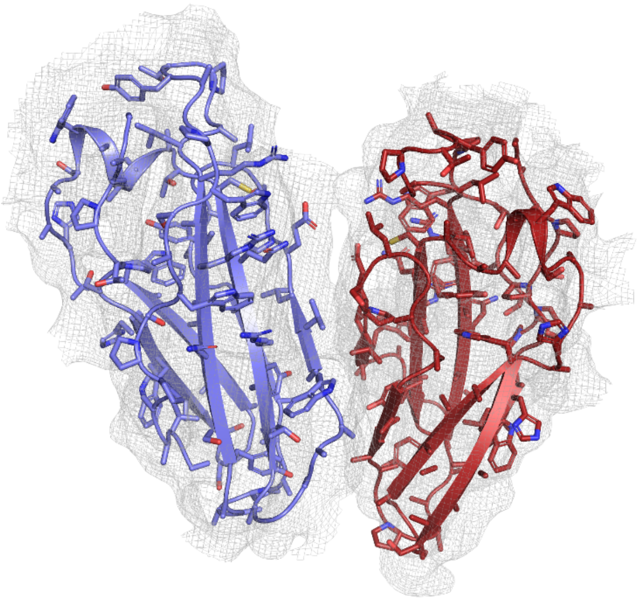

Supplementary Figure 6. The D4-D4 interface. Domain 4 from chain A (blue) and B (red) is shown in cartoon and stick representation with density shown as a mesh contoured at 3σ. This region of the structure was of the lowest resolution, partly due to the fact that flexibility in the structure allows the legs of the receptor to flex at their membrane-proximal end as shown in Supplementary Movie 1.

A

```
                  10        20        30        40        50
mTPOR   1 MPSWALFMVTSCLLLALPNQAQVTSQDVFLLALGTEPLNCFSQTFEDLTCFWDEEEAAPS 60
hTPOR   1 MPSWALFMVTSCLLLAPQNLAQVSSQDVSLLASDSEPLKCFSRTFEDLTCFWDEEEAAPS 60
          ****************  * ***:**** ***  .:***:***:*****************

                  70        80        90       100       110
mTPOR  61 GTYQLLYAYRGEKPRACPLYSQSVPTFGTRYVCQFPAQDEVRLFFPLHLWVKNVSLNQTL 120
hTPOR  61 GTYQLLYAYPREKPRACPLSSQSMPHFGTRYVCQFPDQEEVRLFFPLHLWVKNVFLNQTR 120
          *********  ******** ***:* ********** *:*************** ****

                 130       140       150       160       170
mTPOR 121 IQRVLFVDSVGLPAPPRVIKARGGSQPGELQIHWEAPAPEISDFLRHELRYGPTDSSNAT 180
hTPOR 121 TQRVLFVDSVGLPAPPSIIKAMGGSQPGELQISWEEPAPEISDFLRYELRYGPRDPKNST 180
           *************** :*** ********** ** **********:****** * .*:*

                 190       200       210       220                 230
mTPOR 181 APSVIQLLSTETCCPTLWMPNPVPVLDQPPCVHPTASQPHGP--------APFLTVKGGS 232
hTPOR 181 GPTVIQLIATETCCPALQRPHSASALDQSPCAQPTMPWQDGPKQTSPSREASALTAEGGS 240
          .*:****::******:*  *: . .*** **.:**    .**        *  **.:***

               240       250       260       270       280       290
mTPOR 233 CLVSGLQAGKSYWLQLRSQPDGVSLRGSWGPWSFPVTVDLPGDAVTIGLQCFTLDLKMVT 292
hTPOR 241 CLISGLQPGNSYWLQLRSEPDGISLGGSWGSWSLPVTVDLPGDAVALGLQCFTLDLKNVT 300
          **:**** *:********:***:** **** **:***********::********** **

               300       310       320       330       340        350
mTPOR 293 CQWQQQDRTSSQGFFRHSRTRCCPTDRDPTWEKCEEEE-PRPGSQPALVSRCHFKSRNDS 351
hTPOR 301 CQWQQQDHASSQGFFYHSRARCCPRDRYPIWENCEEEEKTNPGLQTPQFSRCHFKSRNDS 360
          *******::****** ***:**** ** * **:*****  .** *   .***********

                360       370       380       390       400       410
mTPOR 352 VIHILVEVTTAQGAVHSYLGSPFWIHQAVLLPTPSLHWREVSSGRLELEWQHQSSWAAQE 411
hTPOR 361 IIHILVEVTTAPGTVHSYLGSPFWIHQAVRLPTPNLHWREISSGHLELEWQHPSSWAAQE 420
          :********** *:*************** ****.*****:***:******* *******

                420       430       440       450       460       470
mTPOR 412 TCYQLRYTGEGREDWKVLEPSLGARGGTLELRPRARYSLQLRARLNGPTYQGPWSAWSPP 471
hTPOR 421 TCYQLRYTGEGHQDWKVLEPPLGARGGTLELRPRSRYRLQLRARLNGPTYQGPWSSWSDP 480
          ***********::******* *************:** *****************:** *

                530
mTPOR 472 ARVSTGSETA 531
hTPOR 480 TRVETATETA 540
          :**.*.:***
```

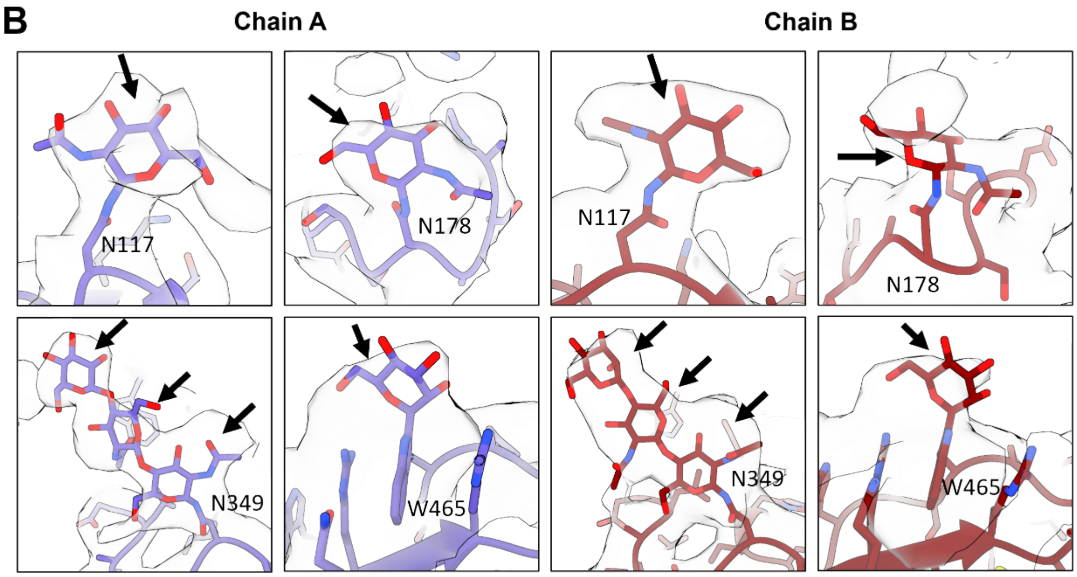

Supplementary Figure 7. Glycosylation of TpoR. (A) Sequence alignment of mouse and human TpoR using Clustal Omega with identified glycosylation sites highlighted: N-linked glycosylation (yellow) and C-mannosylation (cyan). (B) Zooms of cryo-EM reconstruction (transparent) with modelled glycans (sticks) for both chains: N117-NAG, N178-NAG, N349-NAG-NAG-NAG, and W465-MAN.

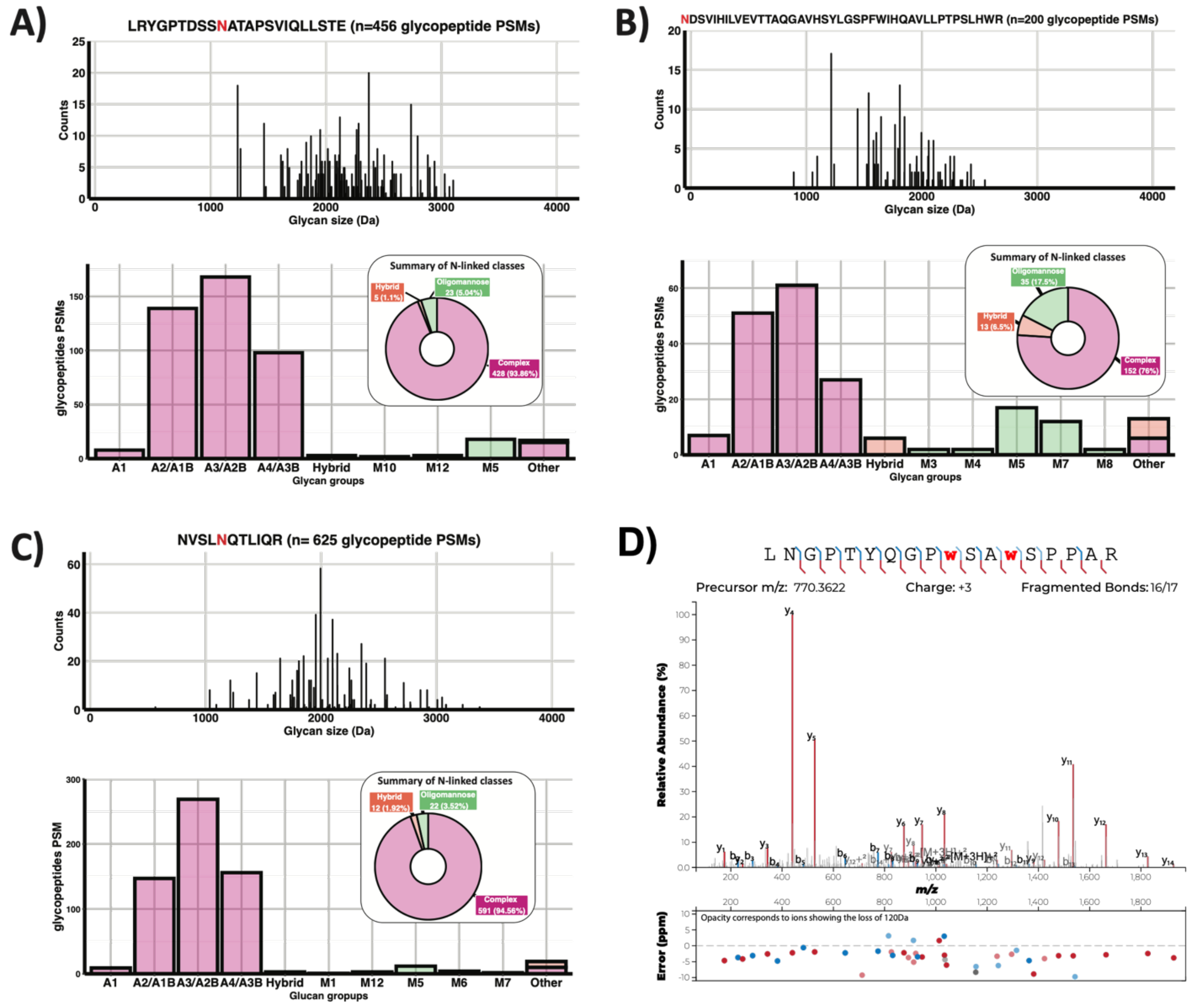

Supplementary Figure 8. Mass spectroscopy analysis of TpoR glycan modifications

(A-C) N-linked glycosylation of N-117, N-178 and N-349, showing complex, low-mannose glycans are the major species.

(D) MS/MS spectrum of an identified mannosylated peptide.

Sequence: LNGPTYQGPWSAWSPPAR (TPOR Man-Trp 465 / Man-Trp 468).

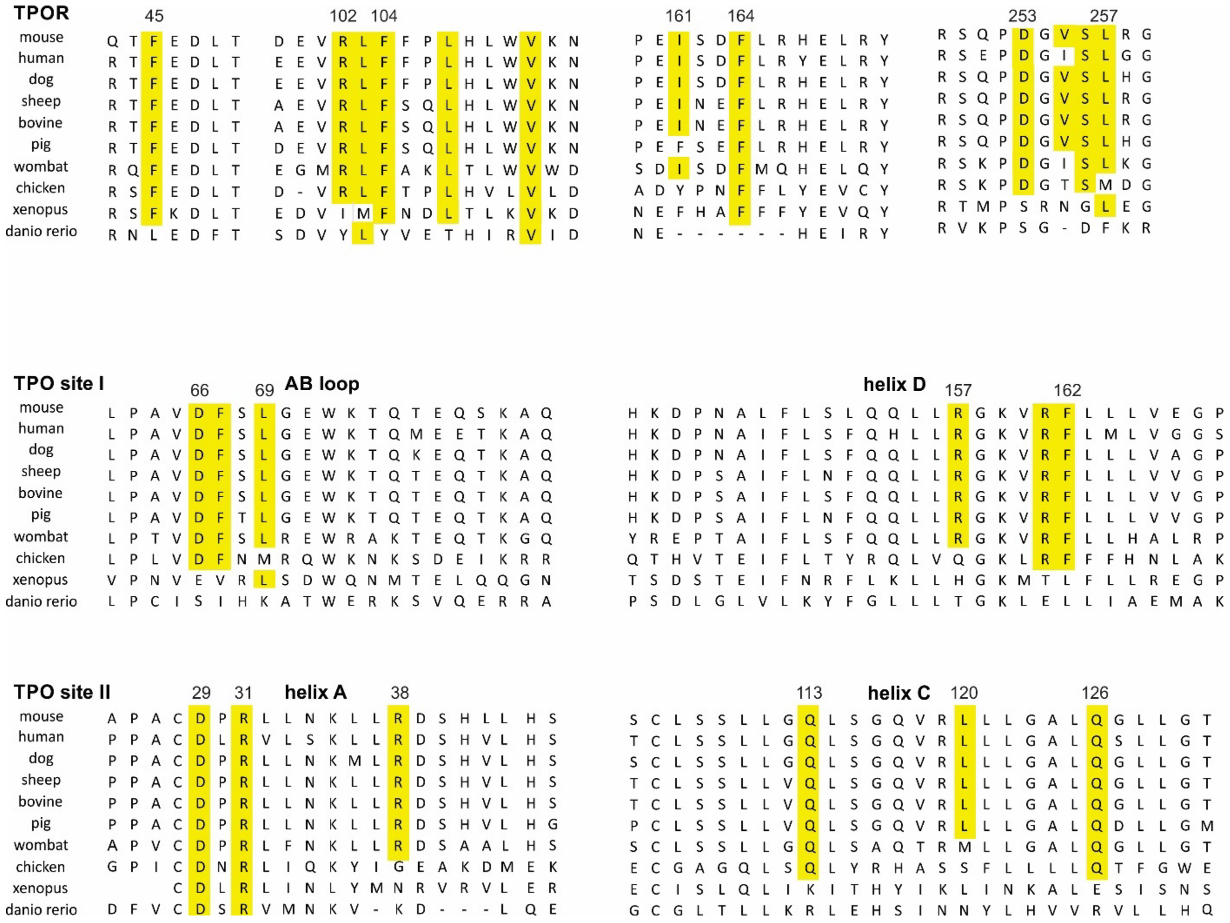

Supplementary Figure 9. Sequence conservation of Tpo and TpoR. Residues referred to in the text are highlighted in yellow.

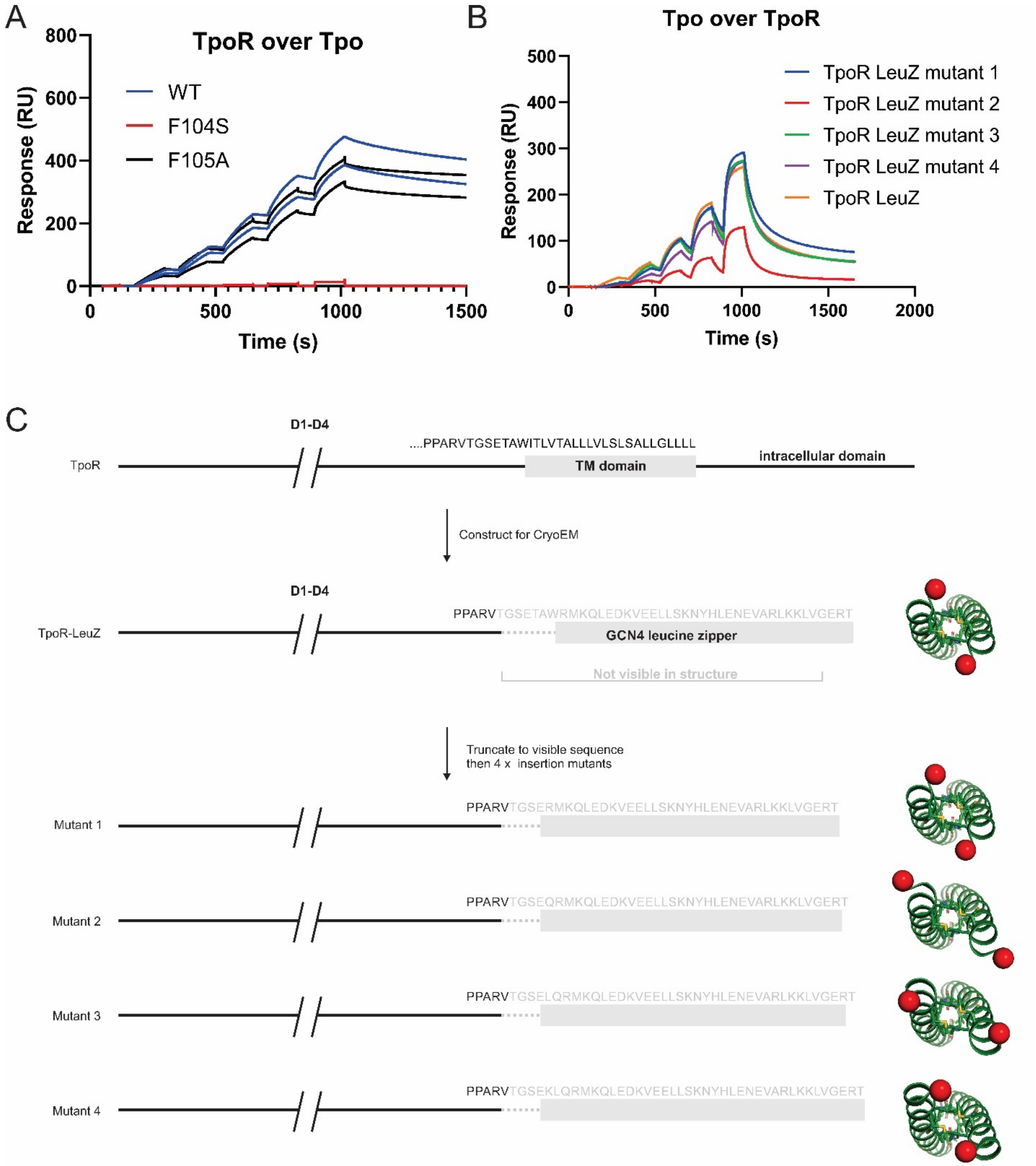

Supplementary Figure 10. SPR evaluation of TpoR F104 mutants and leucine zipper variations. (A) The mutation of F104 (but not F105) dramatically reduces affinity for Tpo. Dimeric (leucine-zippered) TpoR (wild-type, F104S and F105A) was passed over immobilised Tpo. Data are from a single cycle kinetics experiment run in duplicate and is representative of two independent experiments. The top concentration of analyte was 250 nM preceded by four 2-fold dilutions. (B) Altering the helical register of the leucine zipper does not increase the affinity of dimeric TpoR for Tpo. Data are from a single cycle kinetics experiment and is representative of two independent experiments. The top concentration of

analyte was 250 nM preceded by four 3-fold dilutions. (C) Graphic description of the original Leucine zippered construct and the four insertion mutants constructed. The images to the right are a top view of the GCN4 leucine zipper dimer (PDB ID: 2DGC) with the red spheres indicating the positions where the TpoR ectodomains are fused.

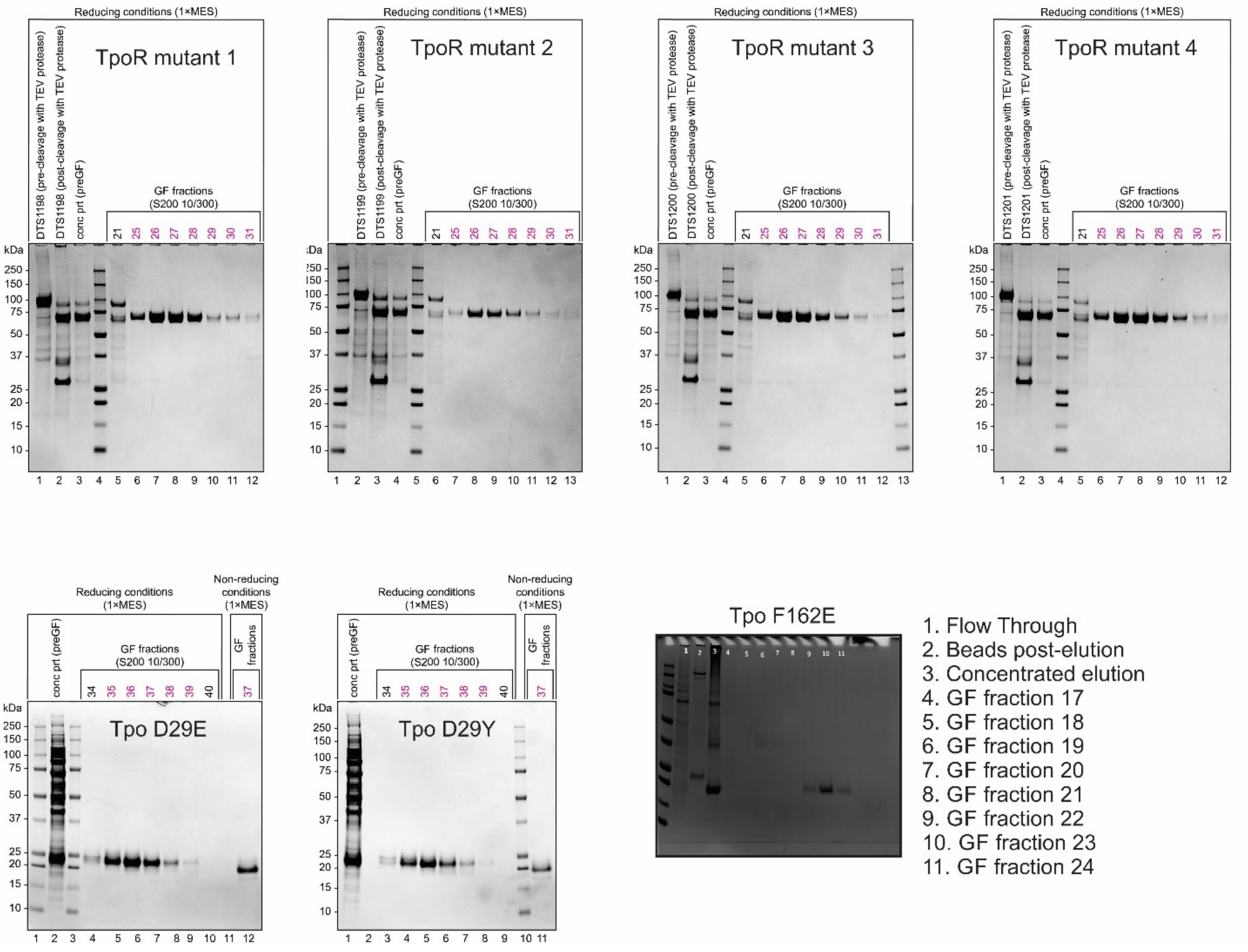

Supplementary Figure 11. Mutant protein purifications. Coomassie stained SDS-PAGE gels of the purification of Tpo and TpoR mutants produced in this study.

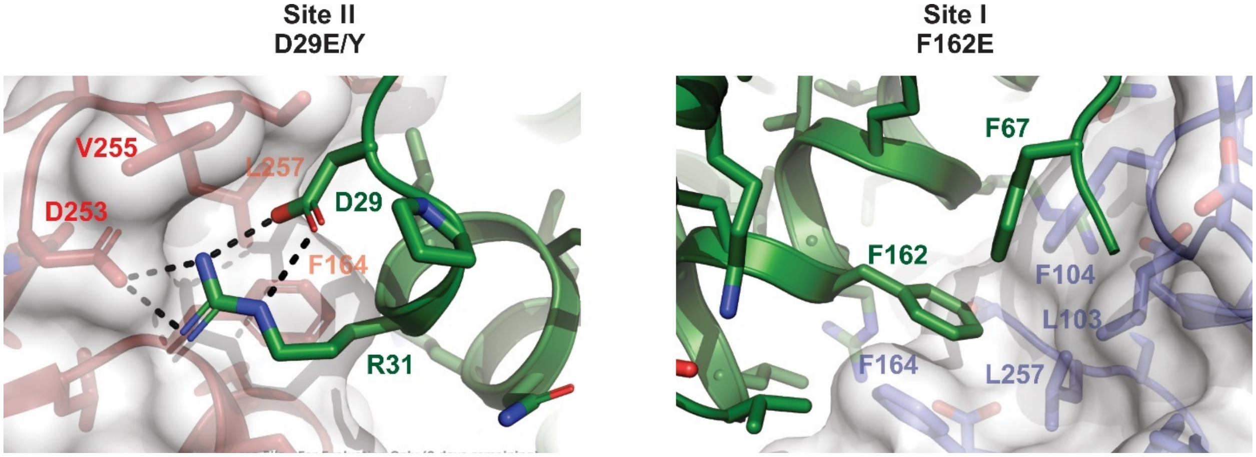

Supplementary Figure 12. Mutation of site I and site II. Left, Site II mutants were constructed by mutating Tpo D29 to Asp and Tyr. The sidechain of D29 points into a hydrophobic pocket formed by TpoR V255, L257 and F164. The extra-length of the Asp and Tyr sidechains would clash with those residues. In addition D29 orients Tpo R31 which hydrogen bonds to TpoR D53. Right, the Site I mutant was constructed by mutating Tpo F162 to Glu. F162 sits in a hydrophobic pocket formed by TpoR F104, L103, L257 and F164. By mutating F162 to a charged residue we aimed to prevent this interaction.

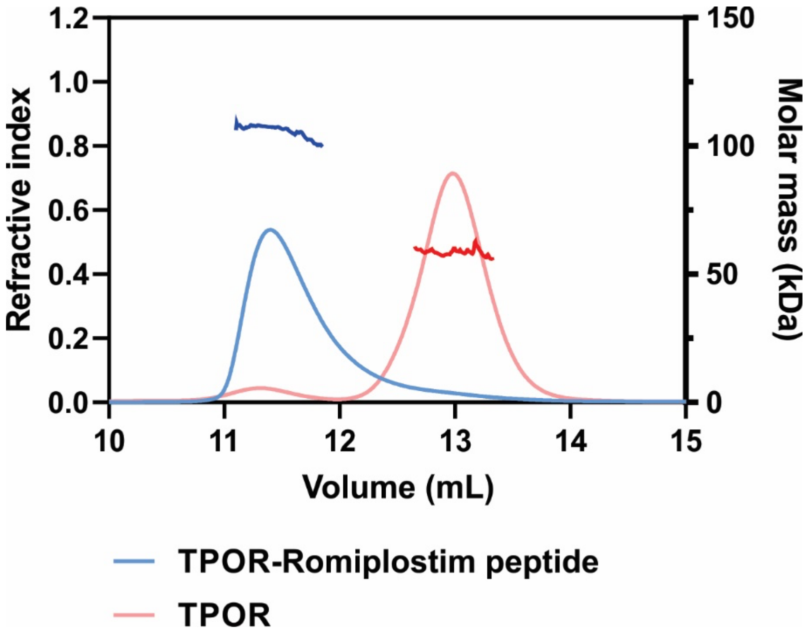

Supplementary Figure 13. SEC-MALS analysis shows that dimeric romiplostim peptide can dimerise TpoR.

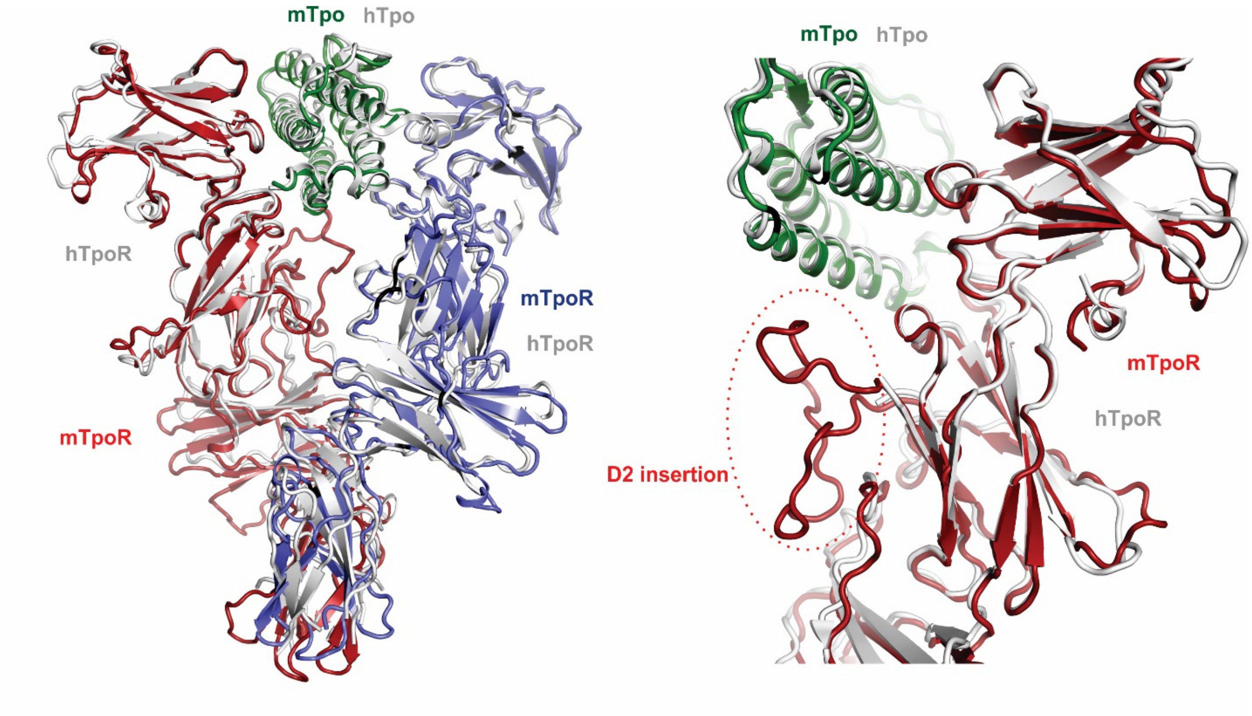

Supplementary Figure 14. A comparison of the mouse and human Tpo:TpoR structures. Left, Overlay of the human Tpo:TpoR complex (white, PDB 8G04) with the murine complex (red, blue, green). mTpo(R) is murine, hTpo(R) is human. The structures overlay with a backbone R.M.S.D of 2 Å. Right, the D2 insertion in the human structure is not of sufficient resolution to be resolved.
