# Supplementary material for: Cryo-EM structure of the extracellular domain of murine Thrombopoietin Receptor in complex with Thrombopoietin
Source: Nat Commun. 2024 Feb 7;15:1135. doi: 10.1038/s41467-024-45356-2 (PMC10850085; doi:10.1038/s41467-024-45356-2)
Supplement: Supplementary file 3 — Description of Additional Supplementary Files [file 41467_2024_45356_MOESM3_ESM.pdf]

Description of Additional Supplementary Files

Supplementary Data 1. LC-MS N-linked and C-linked glycopeptide analysis of TpoR ectodomain.

Supplementary Movie 1. Volume series generated by 3DFlex of Tpo:TpoR reconstruction with three latent dimensions, highlighting the flexibility of D3 and D4 relative to CHR1 and the cytokine.
