# Supplementary material for: Cryo-EM structure of the extracellular domain of murine Thrombopoietin Receptor in complex with Thrombopoietin
Source: Nat Commun. 2024 Feb 7;15:1135. doi: 10.1038/s41467-024-45356-2 (PMC10850085; doi:10.1038/s41467-024-45356-2)
Supplement: Supplementary file 6 — Reporting Summary [file 41467_2024_45356_MOESM6_ESM.pdf]

nature portfolio

Corresponding author(s): Nadia Kershaw and Jeffrey Babon

Last updated by author(s): Dec 19, 2023

Reporting Summary

Nature Portfolio wishes to improve the reproducibility of the work that we publish. This form provides structure for consistency and transparency in reporting. For further information on Nature Portfolio policies, see our Editorial Policies and the Editorial Policy Checklist.

Statistics

For all statistical analyses, confirm that the following items are present in the figure legend, table legend, main text, or Methods section.

n/a	Confirmed	
☐	☒	The exact sample size (*n*) for each experimental group/condition, given as a discrete number and unit of measurement
☐	☒	A statement on whether measurements were taken from distinct samples or whether the same sample was measured repeatedly
☒	☐	The statistical test(s) used AND whether they are one- or two-sided<br>*Only common tests should be described solely by name; describe more complex techniques in the Methods section.*
☐	☒	A description of all covariates tested
☐	☒	A description of any assumptions or corrections, such as tests of normality and adjustment for multiple comparisons
☐	☒	A full description of the statistical parameters including central tendency (e.g. means) or other basic estimates (e.g. regression coefficient) AND variation (e.g. standard deviation) or associated estimates of uncertainty (e.g. confidence intervals)
☒	☐	For null hypothesis testing, the test statistic (e.g. *F*, *t*, *r*) with confidence intervals, effect sizes, degrees of freedom and *P* value noted<br>*Give P values as exact values whenever suitable.*
☒	☐	For Bayesian analysis, information on the choice of priors and Markov chain Monte Carlo settings
☒	☐	For hierarchical and complex designs, identification of the appropriate level for tests and full reporting of outcomes
☒	☐	Estimates of effect sizes (e.g. Cohen's *d*, Pearson's *r*), indicating how they were calculated

Our web collection on statistics for biologists contains articles on many of the points above.

Software and code

Policy information about availability of computer code

Data collection	BIAcore experiments were collected with the help of BIAcore 8K control software v3.0.12.15655.<br>Stability assay data were collected using a Nanotemper Tycho NT.6 and control software v1.3.2.880.<br>Mass photometry data were collected using the Refeyn AquireMP software v2023 1.1.0<br>The CryoEM dataset of 6,130 movies was recorded using EPU 2 (FEI) on a K3 Summit direct electron detector (Gatan Inc., USA)

Data analysis	BIAcore experiments were evaluated using BIAcore Insight evaluation software (v3.0.12.15655). Graphpad prism 9.0 was used to further analyze BIAcore data and determine IC50 and KD values.<br>Stability assay data were collected and analysed using a Tycho NT.6 Nanotemper. Graphpad prism 9.0 was used to further analyze data stability assays. Graphpad prism 9.0 was used to analyze cellular and ec50 assays<br>Mass photometry data were analysed using the DiscoverMP software (v2023 R.1.2)<br>Reconstruction of the TpoR:Tpo structure was performed using cryoSPARC (v.4.3.0)<br>To resolve the density of domains 3 and 4, we used 3D Flex within cryoSPARC (v.4.3.0) and performed local sharpening using DeepEMhancer (v. 0.15)<br>An initial model of TpoR was produced using AlphaFold2 on Google CoLab and fitted into the maps using rigid body fits of each domain in ChimeraX (v. 1.6.1)<br>Modelling was performed in Coot (v. 0.9.8.3)<br>The models were refined in real space with the phenix.real_space_refine program (PHENIX v. 1.20.1_4487)<br>The geometry and quality of the models were evaluated using PHENIX v. 1.20.1_4487<br>Visualisation and analysis of the models and maps were performed using ChimeraX and PyMOL (Schrödinger, LLC, v. 2.5.0)<br>Visualization of proteomic data was undertaken using R (version 4.2.1) and the tidyverse collection of packages.

For manuscripts utilizing custom algorithms or software that are central to the research but not yet described in published literature, software must be made available to editors and reviewers. We strongly encourage code deposition in a community repository (e.g. GitHub). See the Nature Portfolio guidelines for submitting code & software for further information.

Data

Policy information about availability of data

All manuscripts must include a data availability statement. This statement should provide the following information, where applicable:

- Accession codes, unique identifiers, or web links for publicly available datasets
- A description of any restrictions on data availability
- For clinical datasets or third party data, please ensure that the statement adheres to our policy

We have provided the following statement on data availability in the manuscript:
The structural data (atomic coordinates and cryo-EM density maps) generated in this study have been deposited in the Protein Data Bank (PDB) and Electron Microscopy Data Bank (EMDB) databases under accession code 8U18 [https://doi.org/10.2210/pdb8U18/pdb] (murine Tpo:TpoR complex) and EMD-41805 [https://www.ebi.ac.uk/pdbe/entry/emdb/EMD-41805] (murine Tpo:TpoR complex). The structures used for comparison in this study are available in the Protein Data Bank under the following accession codes: 1V7N [http://doi.org/10.2210/pdb1V7N/pdb] (human Tpo); 1EER [http://doi.org/10.2210/pdb1EER/pdb] (human Epo:EpoR complex); 3HHR [https://doi.org/10.2210/pdb3HHR/pdb] (Human GH:GHR complex), 3NPZ [https://doi.org/10.2210/pdb3NPZ/pdb] (Human PRL:rat PRLR); 8G04 [http://doi.org/10.2210/pdb8g04/pdb] (human Tpo:TpoR complex). All mass spectrometry data (RAW files, FragPipe outputs, Rmarkdown scripts, and input tables) have been deposited into the PRIDE ProteomeXchange repository102 with the data set identifier: PXD046926 (Username: reviewer_pxd046926@ebi.ac.uk Password: RU7jrDHM). All other data generated in this study are provided in the Supplementary Information.

Research involving human participants, their data, or biological material

Policy information about studies with human participants or human data. See also policy information about sex, gender (identity/presentation), and sexual orientation and race, ethnicity and racism.

Reporting on sex and gender	N/A
Reporting on race, ethnicity, or other socially relevant groupings	N/A
Population characteristics	N/A
Recruitment	N/A
Ethics oversight	N/A

Note that full information on the approval of the study protocol must also be provided in the manuscript.

Field-specific reporting

Please select the one below that is the best fit for your research. If you are not sure, read the appropriate sections before making your selection.

[x] Life sciences [ ] Behavioural & social sciences [ ] Ecological, evolutionary & environmental sciences

For a reference copy of the document with all sections, see nature.com/documents/nr-reporting-summary-flat.pdf

Life sciences study design

All studies must disclose on these points even when the disclosure is negative.

Sample size	Sample size for each experiment was determined by minimum number required to achieve similar robust results, however for some experiments additional repeats were performed particularly those aimed at quantifying the site I interaction.

For all BIAcore experiments, a minimum of three independent experiments were performed and used to generate the final data. However more than 10 preliminary experiments were performed to determine the minimum concentration of analyte and the maximum flow rate needed to generate robust data. SPR analyses are more robust when the concentration of analyte is low and the flow rate is high so as to avoid non-specific binding and mass-transfer effects and hence these preliminary experiments were not included in the final analyses.
For Mass-photometry data three independent experiments were performed.
For the Tpo/Romiplostim competition experiments two independent experiments were performed each with technical triplicates
Ba/F3 growth assays were performed twice independently, each with technical duplicates.
M1 differentiation assays were performed three times independently, each time with technical duplicates
SECMALS data shown are from two independent experiments however preliminary experiments to optimise the stoichiometry were initially performed and these were omitted from the final analysis.
The number of independent repeat experiments is stated in the figure legend in all cases.

Data exclusions	Some data points were excluded from BIAcore and stability assays due to technical errors such as a damaged flow cell, or no melting curve detected. These datasets or data points were excluded from analysis.
Replication	Biochemical assays were repeated independently at least 2-3 times, with duplicate technical replicates to ensure reproducibility. The spread of data is shown throughout the paper through use of both individual data points along with SD error bars. All repeats were successful (excluding technical errors).
Randomization	Randomization was not relevant to our study as experiments performed were quantitative not qualitative. Human bias is mitigated in these experiments by collection and analysis by computer software.
Blinding	Blinding was not relevant to our study as experiments performed were quantitative not qualitative. Human bias is mitigated in these experiments by collection and analysis by computer software.

Reporting for specific materials, systems and methods

We require information from authors about some types of materials, experimental systems and methods used in many studies. Here, indicate whether each material, system or method listed is relevant to your study. If you are not sure if a list item applies to your research, read the appropriate section before selecting a response.

Materials & experimental systems

n/a	Involved in the study
☒	☐ Antibodies
☐	☒ Eukaryotic cell lines
☒	☐ Palaeontology and archaeology
☒	☐ Animals and other organisms
☒	☐ Clinical data
☒	☐ Dual use research of concern
☒	☐ Plants

Methods

n/a	Involved in the study
☒	☐ ChIP-seq
☒	☐ Flow cytometry
☒	☐ MRI-based neuroimaging

Eukaryotic cell lines

Policy information about cell lines and Sex and Gender in Research

Cell line source(s)	M1 and Ba/F3 cells are from WEHI
Authentication	Cell lines were not genetically authenticated, however their morphology was visually confirmed.
Mycoplasma contamination	PCR and Invivogen PlasmiTest Kit determined cell lines were mycoplasma negative.
Commonly misidentified lines (See ICLAC register)	No commonly misidentified cell lines were used in this study
